# Prediction of Chloride Distribution for Offshore Concrete Based on Statistical Analysis

**DOI:** 10.3390/ma13010174

**Published:** 2020-01-01

**Authors:** Qing-feng Liu, Zhi Hu, Xian-yang Lu, Jian Yang, Iftikhar Azim, Wenzhuo Sun

**Affiliations:** 1 State Key Laboratory of Ocean Engineering, School of Naval Architecture, Ocean & Civil Engineering, Shanghai Jiao Tong University, Shanghai 200240, China; liuqf@sjtu.edu.cn; 2 State Key Laboratory of Structural Analysis for Industrial Equipment, Dalian University of Technology, Dalian 116024, China; z.hu@lboro.ac.uk; 3 School of Architecture, Building and Civil Engineering, Loughborough University, Loughborough LE11 3TU, UK; 4 School of Civil Engineering, University of Birmingham, Birmingham B15 2TT, UK

**Keywords:** chloride, durability, long-term behaviour, offshore concrete, statistical analysis

## Abstract

Chloride-induced corrosion is the main threat to the service life of concrete structures. In order to better investigate chloride distribution in offshore concrete, this study proposed a new prediction model based on statistical analysis as well as a large body of experimental results collected from various sources. A detailed discussion found that the key influential parameters, such as diffusion coefficient (D), surface chloride concentration ($C_{S}$) and penetration depth (x) are all highly time-dependent. The exposure zone, water–cement ratio and service time were also considered as relevant factors. The proposed model is then validated by two alternative tests and the results suggest that it is feasible in predicting the chloride content and penetration depth of concrete structures in a marine environment under chloride attack.

## 1. Introduction

Offshore precast concrete wind turbine towers are preferred over their steel counterparts when the tower height exceeds 85 m [1]. This is mainly due to the more suitable properties that concrete structures possess, e.g., lower maintenance requirements, better dynamic performance and high versatility [2,3]. The hostile marine environment creates many concerns for offshore concrete structures, in particular, corrosion-induced degradation in reinforced or pre-stressed concrete structures. One of the key causes of such degradation is chloride penetration, which may significantly affect the durability performance of the structures during their designed service life [4]. The penetrated chlorides, as well as the corresponding rebar-corrosion initiation, have therefore become an important factor in the durability design of offshore concrete structures.

Considerable efforts have been made by various researchers to investigate the mechanisms of chloride diffusion and penetration [5,6,7,8,9,10,11,12,13]. A numerical method has been developed that is capable of simulating chloride migration on a meso- or micro-scale and explains how chloride penetration takes place in concrete [5,6,7,14,15,16,17,18]. While researchers focus on this fundamental process in-depth, there appears to be a gap in the understanding of the interaction between the environment and concrete structures [19,20]. Experimental studies can provide many first-hand data on the studied problems [21,22]. However, due to the practical constraints of resources, most of these can only study a limited number of affecting factors and the relevant data are often found distributed in a variety of literature [23,24]. Collection of these data can be extracted using appropriate selection and sorting techniques and then used for relevant statistical analysis to derive empirical models to investigate the key influencing factors on chloride diffusion in concrete structures. For example, Dhir et al. [25] reported a decrease in the diffusion coefficient with an increase in the concrete strength. Eglinton [26] stated that the concrete grade may influence the permeability, based on the observation that a lower grade concrete suffered deeper chloride penetration. Zhang [10] revealed that adding silica fume to concrete dramatically enhanced the chloride resistance ability due to its densified internal structure, which was also supported by other researchers [11,27]. An experiment conducted in the Persian Gulf region suggested that the chloride diffusion coefficient and surface concentration varied with different exposure zones, and curing conditions had a significant effect on the chloride resistance [28,29]. However, most of these experiments only draw quantitative or phenomenological conclusions and lack any quantitative models, therefore, they cannot be used in practical durability design. 

In this paper, a new statistical analysis based model is proposed to predict chloride diffusion in offshore concrete structures, based on a large body of experimental results collected from various sources. The model is validated by calculating the minimum cover thickness according to the existing design codes [30]. The developed model is also compared with other existing prediction models to demonstrate its suitability.

## 2. Research Background 

Fick’s second law is the most commonly used equation to quantify chloride diffusion in a maritime environment and is given as follows:

(1) $$\frac{\partial C}{\partial t}=D\frac{\partial^{2}C}{\partial x^{2}}$$

where C(x,t) is the chloride concentration at depth *x* and at time *t*, and D is the diffusion coefficient. If the surface chloride content at time t, C(0,t), equal to the surface chloride concentration, $C_{s}$, and the initial chloride content at depth x is $C\left(x,0\right)=C_{0}$. The analytical solution for the above equation is:

(2) $$C\left(x,t\right)=C_{0}+(C_{s}-C_{0})\left[1-erf\left(\frac{x}{2\sqrt{Dt}}\right)\right]$$

It should be noted that Equation (2) is valid only if the diffusion coefficient D and the surface chloride concentration $C_{S}$ are constant. However, previous research has shown that these two parameters are highly time-dependent [31,32,33]. In reality, capillary pores develop during the maturity process of concrete and micro-cracks grow due to the concrete structure being subjected to various kinds of loads during its service life, such as fatigue load. Both developments will markedly influence the permeability of concrete. Researchers have proposed different approaches to allow calibration for these two parameters. For instance, Markeset and Skjolsvold [34] presented a calibration equation to predict chloride diffusion based on a 30 years field test in Norway as follows,

(3) $$D\left(t\right)=D_{0}(\frac{t_{0}}{t}{)}^{\alpha }$$

where $D_{0}$ is the diffusion coefficient at time $t_{0}$ and α is the age factor. They also performed a numerical study to investigate the sensitivity of the age factor and found that α was the dominant parameter in the service life predictions. Jin et al. [35] proposed a similar calibration-based model for the diffusion coefficient D and suggested a comprehensive equation:

(4) $$D\left(t\right)=k_{e}k_{w/c}k_{FA}k_{SL}k_{c}D_{RCM}(\frac{t_{0}}{t}{)}^{\alpha }$$

where $k_{e}$, $k_{w/c}$, $k_{FA}$, $k_{SL}$, and $k_{c}$ represent the influence of exposure environment, water–cement ratio, fly ash, slag and curing methods, respectively, on the diffusion coefficient. And $D_{RCM}$ is the chloride migration coefficient measured by the Rapid Chloride Migration (RCM) test. Al-Amoudi et al. [36] also established the relationship between the diffusion coefficient and the concrete strength with a calculation equation as follows,

(5) $$D=\frac{a}{{\left(f_{c}\right)}^{b}}$$

where $f_{c}$ is the compressive strength, and a and b are the empirical constants.

For the surface chloride concentration, $C_{s}$ can be accumulated over time, and Ann et al. [37] proposed the following equation to consider the time effect:

(6) $$C_{S}\left(t\right)=C_{S0}+k\sqrt{t}$$

where $C_{S0}$ is the initial surface chloride content and k is a constant. Considering the time effect, Zhao [38] proposed another equation as follows:

(7) $$C_{S}\left(t\right)=C_{S0}+C_{Smax}-rt_{Smax}$$

where $C_{Smax}$ is the constant chloride surface content and r is an empirical coefficient. This study also proposes the calibration coefficients to consider the influence of the water–cement ratio at a given time according to the ‘DuraCrete’ model [39] as follows:

(8) $$C_{S}=A\left(w/c\right)+B$$

where A and B are empirical coefficients and w/c is the water–cement ratio.

The studies reviewed above have made notable progress in examining the two time-dependent parameters (i.e., D and $C_{S}$). Unfortunately, D and $C_{S}$ in these studies are calibrated by focusing specifically on either one or several factors (i.e., age, environmental conditions, concrete strength) and the resulting models are given in different forms, which lack consistency. Note that the diffusion coefficients referred in this study are mainly calculated from two categories, i.e., field tests and laboratory dummy tests. Field tests are mainly based on rapid chloride test (RCT) for the calculation of chloride diffusions on site, whilst the latter one could be divided into diffusion test (e.g., salt ponding test, AASHTO T259 or bulk diffusion test, NT Build 443) and migration test (e.g., rapid chloride permeability test, AASHTO T277 or rapid migration test, NT Build 492). It is also noted that most of these studied factors can be related to the water–cement ratio in some way and hence some literature suggests that the water–cement ratio could be one of the key relevant factors [40]. This will potentially reduce the number of influencing factors that should be included in the model. All the above models assume D and $C_{S}$ to be two independent variables, but by incorporating them into the Fick’s law equation, the chloride content profile along a certain path, x, and at a given time, t, can be determined. By introducing a chloride threshold level, the critical penetration depth at which this value is met can be calculated. Besides, it is also essential to appreciate the effect of chloride binding effect, which has been taken into consideration for the results of chloride concentration and penetration depth in this study.

Fick’s law model provides a rational approach to examining chloride diffusion through concrete. However, the chloride ions transport issue is far more complicated than the diffusion issue. The model may have to rely on the calibration of D and $C_{S}$ to allow for those coupled influencing factors, some of which are not understood or quantifiable.

An alternative simplified approach assumes the chloride content profile decays with the distance from the concrete surface and will reach a negligibly small level at a depth, x, which is defined as the chloride penetration depth. This can be used to determine the cover thickness [41,42]. In the second approach, various influencing parameters are considered together with a focus on the key ones. 

In this study, both models are used to calibrate D, $C_{S}$ and, in particular, x, which has not previously been considered by many researchers. The key influencing factors, i.e., the water–cement ratio and the exposure environment, are analysed together with the time-dependence effect. This study is only limited to Portland cement to minimise the number of variables.

## 3. Method Description

Chloride penetration data can be obtained from laboratory accelerated tests, site inspection or field exposure tests. Laboratory accelerated tests are most suitable for comparative studies but they may not be able to capture the actual behaviour due to the environmental conditions imposed on the laboratory tests are different from field conditions. Site inspection can obtain the true behaviour data, but the varying exposure history is difficult to record, and many other influencing factors such as the cracks resulting from over-loading may add many coupled uncertainties that will be difficult to separate. The field exposure data can, to a certain degree, address these two problems and provide relatively reliable data as well as the measurable exposure conditions. As a result, this paper only selects field exposure data [29,34,36,43,44,45] to calibrate the chloride distribution model based on the analytical solution of Fick’s second law.

The following sections first examine an experimental study program, in which Portland cement slab specimens with a w/c ratio of 0.3 and 0.5, respectively, were humid-cured for 7 days under the ambient temperature before being exposed to a marine environment containing chloride ions at a concentration of 16–21 g/L [43]. This was then followed by the analysis of five groups of experiments in similar tests [29,34,36,44,45], by utilising and normalising them to give a wider range of data for the w/c ratio. These experiments provide data for the four different marine exposure conditions with a w/c ratio ranging from 0.3 to 0.6, and thus they are comparable and suitable for data fitting.

**Figure 1:**
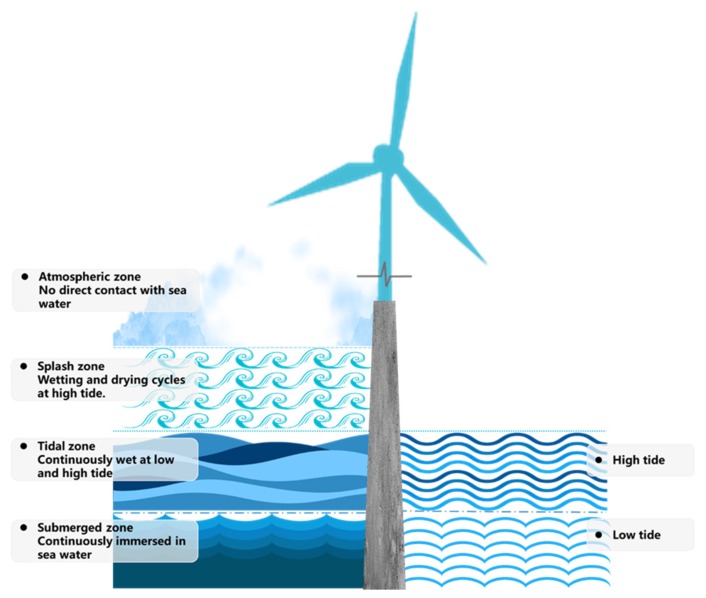
Different exposure conditions.

The data were extracted and reclassified into 4 categories of exposure conditions, including atmospheric zone, splash zone, tidal zone and submerged zone, according to the marine exposure environment (Figure 1). Based on diffusion coefficients, surface chloride concentrations, penetration depths and exposure zones, 416 data sets were adopted corresponding with various exposure zones. Comparison is first made within each individual category to investigate the influence of time and water–cement ratio on D, $C_{S}$ and *x*. Then different groups are combined to examine how the exposure condition affects the chloride penetration.

As well as from seawater, chloride ions may be introduced into concrete as mix contaminants, which are presented as $C_{0}$ in Equation (2). Previous research suggests that the average content of chloride ions in Portland cement is 0.016% [31] and, in this paper, this value is about 0.37 g/L, which is far less than that of seawater 16–21 g/L. $C_{0}$ is, therefore, assumed to be zero in this case and Equation (2) becomes:

(9) $$C\left(x,t\right)=C_{s}\left[1-erf\left(\frac{x}{2\sqrt{Dt}}\right)\right]$$

## 4. Results and Discussion

### 4.1. Diffusion Coefficient of Chloride Ions

**Figure 2:**
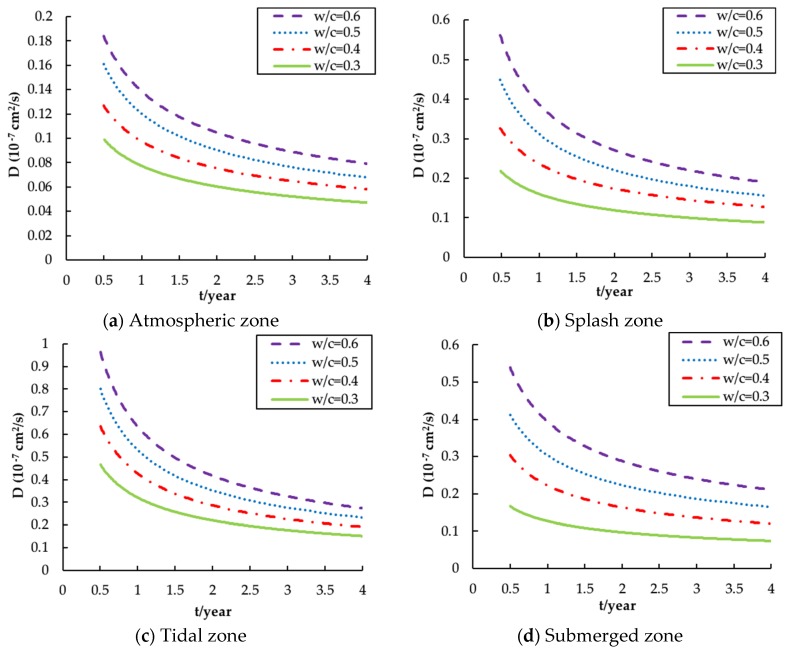
Influence of w/c ratio on chloride diffusion coefficient versus time for different zones: (**a**) Atmospheric zone; (**b**) Splash zone; (**c**) Tidal zone; (**d**) Submerged zone.

The chloride diffusion coefficient (D) is the key variable for indicating the capacity of chloride resistance of a concrete structure. The diffusion coefficient D plotted against exposure time under different w/c ratios in four different exposure zones is presented in Figure 2 [29,36,42,43].

It can be seen clearly that for a given w/c ratio, the chloride diffusivity gradually decreases with the exposure time for all exposure conditions. This is because the hydration continues in the cement binder. Note that the hydration reaction happens not only during the early stage but also after being in service, which leads to a reduction in concrete porosity and thus results in a decrease in diffusivity. It may also be noted that the slope of each curve decreases over time, which indicates that D will eventually reach a relatively constant value when the hydration reaction slows down.

Figure 2 also shows that a larger value of D is always associated with a larger w/c ratio. This is because, at a certain stage of hydration, there is a positive correlation between the w/c ratio and the porosity, the significant indicator of the permeability of concrete [46]. Besides, the w/c ratio is also negative correlation with strength of concrete. It is well-known that the lower strength concrete means lower dense and then lower resistance to chloride and moisture penetration [47]. Interestingly, the curves for various w/c ratios in the same zone are almost parallel to each other. This suggests that the w/c ratio affects the magnitude of D more than the rate of change of D. This observation implies that the w/c ratio has a significant influence on the initial degree of hydration but little influence on the subsequent hydration rate [48].

The exposure environment is another relevant influential factor relating to the diffusion coefficient. As shown in Figure 2, the tidal zone has the highest value of D, followed by the splash zone, the submerged zone and the atmospheric zone, which means that the diffusion coefficient is more than a material property. The tidal and splash zones have a higher value of D because these two external environmental conditions can provide a modest amount of air, moisture and the drying–wetting processes which promote chloride diffusion. This result also partly demonstrates why the drying–wetting processes are identified as the most serious environmental conditions for reinforced concrete durability, which is also contributed by the sorptivity of concrete [49]. It can be seen that the diffusion coefficient in the tidal zone is more than 5 times that in the atmospheric zone, which strongly suggests its significance in the durability design of marine concrete structures.

(10) $$D_{\left(atmosphere\right)}=\left[\left(0.206\right)\left(w/c\right)+\left(0.015\right)\right]\left(10^{-7}\right)t^{-0.388}$$

(11) $$D_{\left(splash\right)}=\left[\left(0.757\right)\left(w/c\right)-\left(0.067\right)\right]\left(10^{-7}\right)t^{-0.472}$$

(12) $$D_{\left(tidal\right)}=\left[\left(1.046\right)\left(w/c\right)+\left(0.007\right)\right]\left(10^{-7}\right)t^{-0.580}$$

(13) $$D_{\left(submerge\right)}=\left[\left(0.879\right)\left(w/c\right)-\left(0.134\right)\right]\left(10^{-7}\right)t^{-0.432}$$

### 4.2. Surface Chloride Concentration

**Figure 3:**
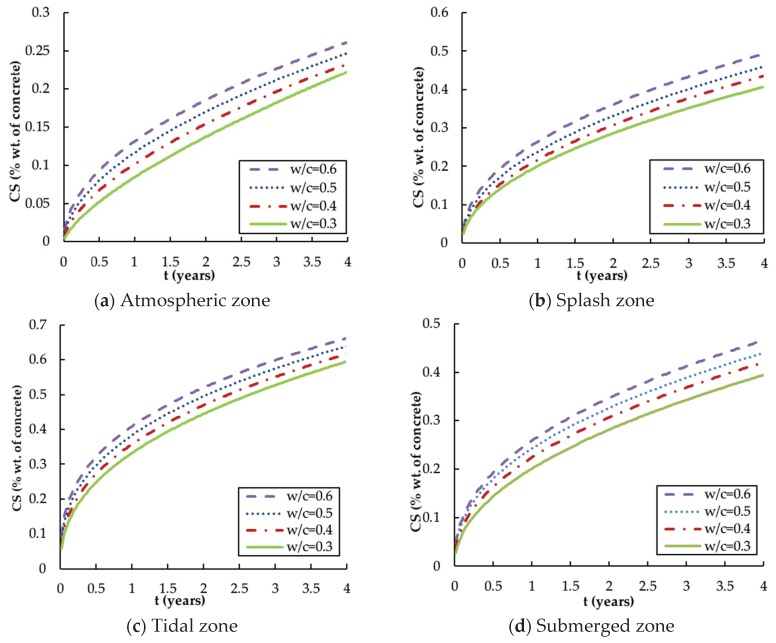
Influence of w/c ratio on surface chloride content versus time for different zones: (**a**) Atmospheric zone; (**b**) Splash zone; (**c**) Tidal zone; (**d**) Submerged zone.

The surface chloride concentration ($C_{S}$) is also an important parameter as it is found that a higher concentration difference between internal and external spaces will lead to higher chloride diffusion into the concrete [38]. Figure 3 shows the development of $C_{S}$ of the specimens with different w/c ratios for four different exposure zones [42,43,44,46].

As expected, the surface chloride content steadily accumulates with time in all exposure conditions, which seems logical for the concrete structure exposed to marine environments in the long term. This confirms the suggestion that $C_{S}$ is a highly time-dependent parameter.

The water–cement ratio has a similar effect on the surface chloride content as it has on the diffusion coefficient, i.e., the values of the surface chloride concentration in different zones slightly increase with w/c ratio and their upward rates become approximately uniform during the latter part of the observed period. The usual view is that $C_{S}$ should be independent of the material properties of the specimen, such as the w/c ratio, as it represents the surface chloride concentration. A reasonable explanation for this discrepancy is that the $C_{S}$ values measured by the core sampling test do not reflect the exact value of the surface chloride content, but only an average value of the chloride concentration within a certain thickness (normally 5 mm) below the concrete surface due to the limitations of the test method [43]. It is noted that this effect associated with the w/c ratio is much smaller than that associated with the service time, with a variation of only about 0.05–0.08%.

The exposure environment also has a much greater influence on $C_{S}$ than the w/c ratio does. According to Figure 3, $C_{S}$ in the tidal zone rises from 0% to (0.60–0.65%) for different w/c ratios, which is a higher value than that in the splash zone (0.40–0.50%) and the submerged zone (0.40–0.45%), and far higher than that in the atmospheric zone (0.23–0.26%). The constant drying and wetting processes in the tidal zone and splash zone, and the sustained direct contact with seawater in the submerged zone are favourable conditions for the accumulation of chlorides on the surface. On the other hand, the chloride ions in the atmospheric zone mainly come from salty moisture in the air and the amount is smaller, which makes it the least affected area.

Previous research suggests that a linear or power function should be used to quantify the relationship between the surface chloride content Cs (%) and the time t (in years) for each exposure zone [50]. The following equations are obtained using a power function which can achieve higher values of the coefficient of determination (R^2^). The higher R^2^ near one in mathematics indicates that the regression predications perfectly fit the data.

(14) $$C_{s\left(atmosphere\right)}=\left[0.156\left(w/c\right)+0.038\right]t^{0.583}$$

(15) $$C_{s\left(splash\right)}=\left[0.213\left(w/c\right)+0.134\right]t^{0.484}$$

(16) $$C_{s\left(tidal\right)}=\left[0.257\left(w/c\right)+0.254\right]t^{0.383}$$

(17) $$C_{s\left(submerge\right)}=\max\{\left[0.193\left(w/c\right)+0.144\right]t^{0.449},C_{seawater}\}$$

where $C_{seawater}$ is the chloride content in the seawater.

Note that in Equation (17), $C_{S}$ has two possible values: $C_{S\left(submerged\right)}$ = [0.191(w/c)+0.146] and $C_{S\left(submerged\right)}$=$C_{seawater}$. This is because the increasing surface chloride concentration will eventually reach a limit which is equal to the chloride content in the seawater. It is also notable that the differences in the coefficients of the w/c ratio term in Equations (14)–(17) are much smaller than those in Equations (10)–(13), which further demonstrates that the exposure is the more dominant factor.

### 4.3. Penetration Depth

**Figure 4:**
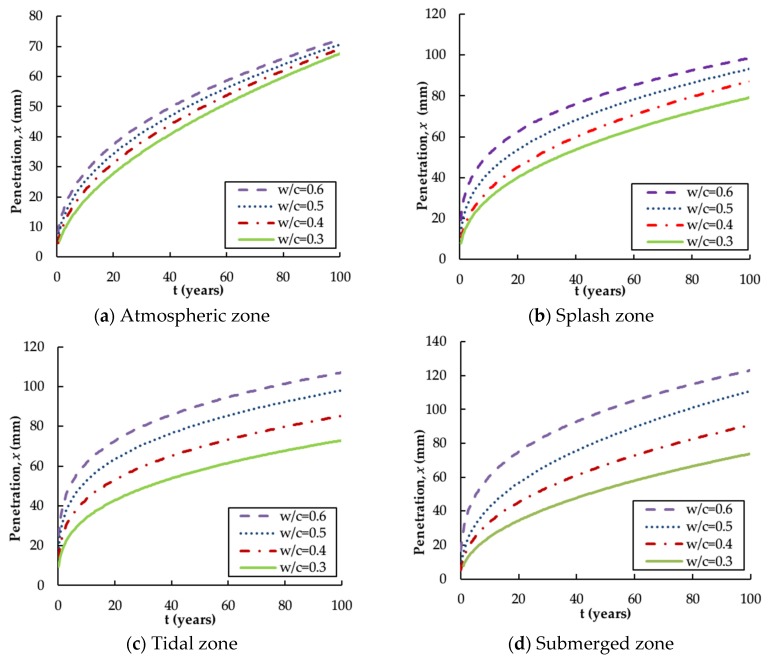
Influence of w/c ratio on penetration depth versus time in different zones: (**a**) Atmospheric zone; (**b**) Splash zone; (**c**) Tidal zone; (**d**) Submerged zone.

Concrete structures exposed to different environmental conditions should have different concrete cover thicknesses [35,36] to prevent steel bars being corroded. The minimum cover thickness of a reinforced concrete structure is set to exceed the penetration depth. Figure 4 presents the penetration depth *x* for different w/c ratios at a specific time in the various environmental zones [29,34,36,46].

Based on the figures, it is clear that x increases dramatically with an increase in the w/c ratio, but the increase tends to be attenuated over time and a higher w/c ratio still leads to higher penetration depth. This follows the same trend as that demonstrated by the chloride diffusion coefficient, D, and the surface chloride content, $C_{S}$, and the reasons for this trend have been discussed previously.

Figure 4 also reveals that the curves have higher discrepancy when exposed to a more extreme marine environment (i.e., the submerged and the tidal zone). This can also be explained by using Fick’s law, that is, higher D and $C_{S}$ values in a severe marine environment amplify the sensitivity of the w/c ratio influence on the chloride penetration rate. In the submerged zone, non-saturated concrete will first take in the salt solution by capillary force; chloride ions then penetrate the concrete by diffusion, which is sometimes accelerated by an increased hydraulic pressure [51]. In other words, the transport of chloride ions in the submerged zone is a mixed mode of capillary suction, diffusion and permeability, which thus leads to the highest penetration rate. In the tidal zone and splash zone, the ingress of chloride ions into the concrete is mainly by capillary suction and diffusion. The depth of penetration primarily depends on there being an excess of water at the concrete surface, and on the duration of this contact with water. The surface concrete in the tidal zone is continuously wet during its service life, whereas in the splash zone it is wet only at high tide. The penetration rate is, therefore, higher in the tidal zone. In the atmospheric zone, capillary suction is the governing transport mechanism, and the lack of sufficient water and chloride ions results in the lowest penetration rate. This explanation also demonstrates why the submerged zone has the highest penetration depth, followed by the tidal zone, the splash zone and then the atmospheric zone as shown in Figure 4.

Similarly, the following relationships can be deduced based on the trends shown in Figure 4. As can be seen, the polynomial function is applied to the coefficient in the splash and the submerged zones for higher accuracy.

(18) $$x_{\left(atmosphere\right)}=\left[18.939\left(w/c\right)-0.423\right]t^{0.476}$$

(19) $$x_{\left(splash\right)}=[139.500\left(w/c{)}^{2}-72.710\left(w/c\right)+20.377\right]t^{0.364}$$

(20) $$x_{\left(tidal\right)}=\left[64.920\left(w/c\right)-3.839\right]t^{0.284}$$

(21) $$x_{\left(submerge\right)}=[238.775\left(w/c{)}^{2}-146.210\left(w/c\right)+31.176\right]t^{0.408}$$

Note that the units of x are mm and those of t are years.

### 4.4. Empirical Formula

The substitution of Equations (10)–(13) and Equations (14)–(17) into the Fick’s second law formula leads to an empirical formula as presented in Equation (22), where the coefficients have been re-labelled. Similarly, the empirical equations for x in Equations (18)–(21) are rearranged in Equation (23) using the relabelled coefficients. The empirical coefficients $a_{1}$ to $a_{10}$ are summarised and listed in Table 1 according to the four exposure conditions.

(22) $$C\left(x,t\right)=\left[a_{1}\left(w/c\right)+a_{2}\right]t^{a_{3}}\times \left[1-erf\frac{x}{112313\sqrt{\left[a_{4}\left(w/c\right)+a_{5}\right]t^{a_{6}}}}\right]$$

(23) $$x=[a_{7}\left(w/c{)}^{2}+a_{8}\left(w/c\right)+a_{9}\right]t^{a_{10}}$$

where C(x,t) is the chloride ion distribution profile at time t. The units of t, x and C(x,t) are year, mm and percentage, respectively. Regarding the term of the surface chloride concentration, $\left[a_{1}\left(w/c\right)+a_{2}\right]t^{a_{3}}$, it should be noted that for the submerged zone, its value should not exceed the chloride concentration of seawater. Equation (22), together with the coefficients listed in Table 1, can be used to predict the chloride profile at any time *t*. Equation (23) can be used to predict the critical chloride penetration depth by using the empirical formula directly.

Rearranging Equation (22), the following formula can be deduced:

(24) $$x_{critical}=112313\sqrt{\left[a_{4}\left(w/c\right)+a_{5}\right]t^{a_{6}}}\times erf^{-1}\left\{1-\frac{C_{critical}}{\left[a_{1}\left(w/c\right)+a_{2}\right]t^{a_{3}}}\right\}$$

where $x_{critical}$ is the critical depth of chloride penetration (mm) and $C_{critical}$ is the chloride threshold.

## 5. Comparison and Validation

To provide a certain level of confidence, the introduced empirical equations, Equation (22) and Equation (23), are validated using two alternative methods. One method is to compare the minimum cover thickness using calculated penetration depth with that required in the specification [30] to affirm its feasibility in structural design. The other method is to make a comparison between the present empirical equation (Equation (22)) and another existing calibration model in predicting the chloride distribution for the same experiment.

### 5.1. Minimum Cover Thickness

Durability in the offshore environment is most commonly related to the resistance to corrosion of reinforced concrete structures, where the protection of the reinforcement plays an important role [52,53,54,55]. The durability requirements provided in the marine concrete specification UFGS-03 [38] indicate a minimum cover thickness for different exposure zones that are summarised in Table 2.

The marine concrete specification UFGS-03 also stipulates the maximum w/c ratio should not exceed 0.4, and the AASHTO [56] guidelines typically state 75 years as a design life for reinforced concrete structures. The chloride threshold is generally regarded as 0.4% by weight of cement [57], whereas $C_{critical}$ in this paper refers to the weight of binder and this value should be 0.05%. Using the values: 0.4, 75 and 0.05% for w/c, t and $C_{critical}$, respectively in Equation (24) and adopting different coefficients from Table 1, the critical depths can be derived as shown in the following table:

The calculated values in Table 3 show that the critical depths of the splash, tidal and submerged zones are quite close to each other and are significantly higher than those of the atmospheric zone. This coincides with the explanatory statement in the previous section.

Likewise, by substituting w/c = 0.4 and t = 75 into Equation (23), the penetration depth, x for each zone can be obtained as listed in Table 4.

Most of the values in Table 4 are marginally higher than those in Table 3, except the exposure zone ‘Submerged’. This is because Equation (24) considers a non-zero chloride threshold, while Equation (23) considers the near zero chloride situation. As a consequence, the results in Table 3 can be regarded as providing an economic solution while those in Table 4 are more conservative. It should be noted that due to the computational errors and also sometimes physical truth, the submerged exposure zones will not always result in higher chloride penetrations.

It can be seen that all the calculated values in Table 3 and Table 4 are in good agreement with the cover thickness requirements in UFGS-03 31 29, as none of them exceeds the corresponding figures in Table 2.

### 5.2. Comparison and Validation with Existing Models

In previous research, Liu et al. [50] presented a quantified model (Equation (25)) based on their laboratory test results as follows:

(25) $$C\left(x,t\right)=C_{0}+\left(C_{s}-C_{0}\right)\left[1-erf\left(\frac{x}{2\sqrt{\frac{k_{e}D_{0}t_{0}^{a}t^{1-a}}{1-a}}}\right)\right]$$

where $k_{e}$ is the environmental coefficient, $D_{0}$ is the chloride diffusion coefficient at time $t_{0}$ and a is the empirical coefficient, i.e., a = 3 × (0.55 − w/c)

The calibration method in Equation (25) is different from that in the empirical formula used in Equation (22). It introduces only one coefficient, $k_{e}$ to consider the influence of different exposure zones, which is more practical than classifying the empirical coefficients into four groups (Table 1). Moreover, it represents the time-dependency of D, and incorporates the w/c ratio as the affecting factor. However, it should be noted that this model also recognises exposure zone, w/c ratio and time as the main affecting factors, which correlates well with the findings of this paper.

**Figure 5:**
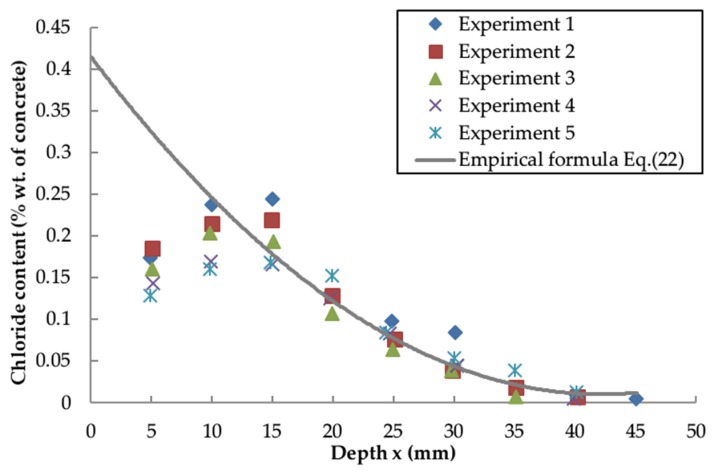
Comparison between experimental results and the results by presented formula (Equation (22)).

**Figure 6:**
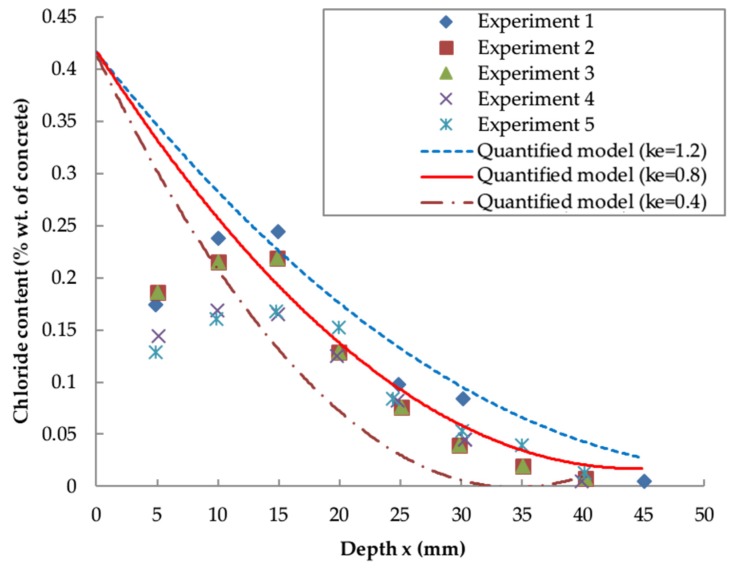
Comparison between experimental results and quantified models.

Figure 5 and Figure 6 compare the predicted chloride profiles obtained from the empirical formula (Equation (22)) and quantified model from Liu et al. [57] (Equation. (25)), with the experimental results for concrete of 0.5 w/c ratio exposed in a splash zone for 36 months [43].

Figure 5 suggests that there is a good agreement between the empirical formula and the experimental results, particularly at the greater depth. The variation at x = 5 mm is probably caused by the effect of surface leaching or anomalies in testing. This demonstrates the feasibility of the empirical formula Equation (24) together with the empirical coefficients (Table 1) in predicting the local chloride distribution. According to Figure 6, the results from the quantified model [57] highly depends on the value of $k_{e}$ indicating the influence of the local environment. In this study, $k_{e}$ = 0.4 and $k_{e}$ = 1.2 represent the lower and upper bounds of the chloride concentration, respectively, and the average value $k_{e}$ = 0.8 is deemed to be the optimum coefficient.

**Figure 7:**
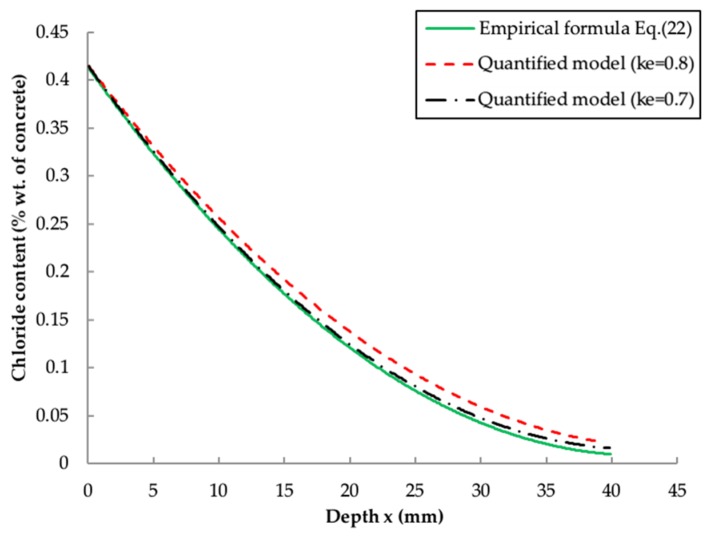
Comparison between empirical formula (Equation (22)) and quantified model (Equation (25)).

Figure 7 shows a comparison between the empirical formula (Equation (22)) and quantified model (Equation (25)). It is evident that the curves are quite close to each other, suggesting that the chloride profiles obtained from these two models are similar. But it is noteworthy that the dash-dot line ($k_{e}$ = 0.7) is much closer to the solid line (empirical formula) than the dashed line ($k_{e}$ = 0.8). This result indicates one of the disadvantages of the quantified model that it is difficult to determine the optimum value of $k_{e}$, especially when there is a large amount of data. Another disadvantage is that there is no calibration expression for $C_{S}$, which makes it hard to calculate the chloride profile in the initial stage. In this example, the value of $C_{S}$ is calculated using Equation (9). In this context, the empirical formula developed in this paper may be simpler and more practical in predicting the chloride content.

## 6. Conclusions

This paper has proposed a new quantified model to investigate chloride diffusion in offshore concrete structures. Based on an investigation into the influence of exposure zones, water–cement ratio and time, the following conclusions can be drawn:

- D, $C_{S}$ and x are highly time-dependent parameters and the calibration of these influence parameters is necessary for the reliable prediction of chloride distribution. The revised D, $C_{S}$ and *x* are functions of exposure zone, w/c ratio and service time.
- The tidal zone is the most severe exposure environment in terms of chloride corrosion, followed by the splash zone, the submerged zone and the atmospheric zone. This affirms that the aggressiveness of the offshore environment varies considerably with the exposure zones and hence the cover thickness should be varied as appropriate for different environments.
- The water–cement ratio is highly relevant to chloride diffusion in that the higher w/c ratio will lead to a greater diffusion coefficient, surface concentration and penetration depth. Thus, it is reasonable to limit the maximum w/c ratio of offshore concrete structures.
- The validity of the proposed empirical formula is confirmed by a successful prediction of a series of independent experiments and the calculated cover thickness is in good agreement with the code UFGS-03. The approach developed in this paper, therefore, provides a simple but reliable means for examining chloride diffusion in offshore structures.

Finally, it should be stressed that the values of the empirical coefficient are based on particular experimental data. Thus, to predict the chloride distribution in a given structure at a particular place, the empirical coefficients ($a_{1}$–$a_{10}$ in Table 1) should be revised accordingly.

## 7. Recommendation for Future Work

This research work investigates the chloride diffusion in Portland cement, considering the influence of w/c ratio, exposure zone and service time. However, as previously stated, researchers have also found that other factors, such as curing methods, concrete grade and the inclusion of cementitious additives may possibly affect the concrete chloride resistance. Therefore, investigations into all the affecting factors would be beneficial for developing a more general empirical formula, which should have a more extensive application but would require a lot more research.

## Figures and Tables

**Table 1:** Empirical coefficient for different exposure zones.

Exposure Zone	Empirical Coefficients									
	$a_{1}$	$a_{2}$	$a_{3}$	$a_{4}$	$a_{5}$	$a_{6}$	$a_{7}$	$a_{8}$	$a_{9}$	$a_{10}$
Atmospheric	0.16	0.04	0.58	0.20 × 10^−7^	0.02 × 10^−7^	0.61	0	18.94	−0.43	0.48
Splash	0.21	0.13	0.48	0.76 × 10^−7^	−0.01 × 10^−7^	0.53	139.50	−72.71	20.38	0.36
Tidal	0.26	0.25	0.38	1.05 × 10^−7^	0.01 × 10^−7^	0.42	0	64.92	−3.84	0.28
Submerged	0.19	0.14	0.45	0.88 × 10^−7^	−0.13 × 10^−7^	0.57	238.78	−146.21	31.18	0.41

**Table 2:** Cover requirements from NAVFAC [30] marine concrete specification.

Zone	Cover Thickness (mm)
Atmospheric zone not subject to salt spray	65
Tidal, splash and atmospheric zone subject to salt spray	75
Submerged zone	75

**Table 3:** Critical depth calculated using Equation (24).

Exposure Zone	Critical Depth(mm)
Atmospheric	50
Splash	64
Tidal	68
Submerged	67

**Table 4:** Penetration depth calculated using Equation (23).

Exposure Zone	Critical Depth(mm)
Atmospheric	56
Splash	66
Tidal	75
Submerged	64
